# SNAP25 is a potential prognostic biomarker for prostate cancer

**DOI:** 10.1186/s12935-022-02558-2

**Published:** 2022-04-07

**Authors:** Longjiang Di, Maoli Gu, Yan Wu, Guoqiang Liu, Lishuo Zhang, Yifei Li, Wenjing Zhang

**Affiliations:** 1 Department of Clinical Laboratory, Second Affiliated Hospital of Harbin Medical University, Harbin, 150086 Heilongjiang China; 2 Department of Urology, The Second Affiliated Hospital of Harbin Medical University, Harbin, 150086 Heilongjiang China; 3 Department of Ultrasound, The Second Affiliated Hospital of Harbin Medical University, Harbin, 150086 Heilongjiang China; 4 Department of Neurosurgery, The Second Affiliated Hospital of Harbin Medical University, Harbin, 150086 Heilongjiang China

**Keywords:** Synaptosome associated protein 25, Chemokines, Migration, Prostate cancer

## Abstract

**Background:**

Prostate cancer (PCa) is one of the most lethal cancers in male individuals. The synaptosome associated protein 25 (*SNAP25*) gene is a key mediator of multiple biological functions in tumors. However, its significant impact on the prognosis in PCa remains to be elucidated.

**Methods:**

We performed a comprehensive analysis of the Cancer Genome Atlas dataset (TCGA) to identify the differentially expressed genes between PCa and normal prostate tissue. We subjected the differentially expressed genes to gene ontology analysis and Kyoto Encyclopedia of Genes and Genomes functional analysis, and constructed a protein–protein interaction network. We then screened for pivotal genes to identify the hub genes of prognostic significance by performing Cox regression analysis. We identified SNAP25 as one such gene and analyzed the relationship between its expression in PCa to poor prognosis using GEPIA interactive web server.

**Results:**

TCGA database demonstrated that SNAP25 was significantly downregulated in PCa. The progressive decrease in SNAP25 expression with the increase in the clinical staging and grading of PCa demonstrates that reduced SNAP25 expression considerably exacerbates the clinical presentation. Our findings confirm that SNAP25 expression strongly correlates with overall survival, which was determined using the Gleason score. We also validated the role of SNAP25 expression in the prognosis of patients with PCa. We used Gene Set Enrichment and Gene Ontology analyses to evaluate the function of SNAP25 and further explored the association between SNAP25 expression and tumor-infiltrating immune cells using the Tumor Immune Assessment Resource database. We found for the first time that SNAP25 is involved in the activation, differentiation, and migration of immune cells in PCa. Its expression was positively correlated with immune cell infiltration, including B cells, CD8^+^ T cells, CD4^+^ T cells, neutrophils, dendritic cells, macrophages, and natural killer cells. SNAP25 expression also positively correlated with chemokines/chemokine receptors, suggesting that SNAP25 may regulate the migration of immune cells. In addition, our experimental results verified the low expression of SNAP25 in PCa cells.

**Conclusion:**

Our findings indicate a relationship between SNAP25 expression and PCa, demonstrating that SNAP25 is a potential prognostic biomarker due to its vital role in immune infiltration.

**Supplementary Information:**

The online version contains supplementary material available at 10.1186/s12935-022-02558-2.

## Background

Prostate cancer (PCa) is the most common cancer in male individuals worldwide, with peak incidence at 70–79 years of age, and is the second most deadly cancer among male individuals in the United States, after lung cancer [1]. Androgen deprivation therapy remains the most common first-line treatment for patients with advanced PCa, but numerous patients with metastatic PCa develop castration resistance post-therapy, which is inextricably linked to their high mortality rate [2, 3]. The pathogenesis of PCa is complicated and involves a large number of genetic aberrations [4]. In recent years, the tumor microenvironment (TME) has been reported to be associated with the progression of PCa [5–7]. Immunotherapy is a promising strategy that has shown significant anticancer effects in PCa [8, 9], and tumor-infiltrating immune cells have been shown to affect patient prognosis as well as the antitumor effects of immunotherapy [10–13]. However, the molecular immune mechanisms associated with PCa remain unclear. Therefore, the identification of new therapeutic biomarkers, specifically ones that are closely associated with PCa immune infiltration, is an urgent need. The ability to correlate a quantitative physiological characteristic with the risk or progression of PCa is essential for early diagnosis and improved prognosis.

Synaptosome associated protein 25 (SNAP25) is a soluble, N-ethylmaleimide-sensitive factor attachment protein receptor complex that is essential for the regulation of neurotransmitter release, synaptic messaging, secretory vesicle extravasation, intercellular signaling, and ion channel opening [14–16]. SNAP25 has been seen as potentially important for normal vesicle fusion and lysosomal trafficking [17, 18]. In recent years, a growing number of studies have identified an association between SNAP25 levels and gastric cancer [19], colon cancer [20], hepatocellular carcinoma [21], lung cancer [22], and papillary thyroid cancer [23]. However, the functions and mechanisms associated with SNAP25 activity in the immunologic and pathogenetic progression of PCa remain to be explored.

With the rapid development of high-throughput sequencing technologies, the mechanistic exploration of a large number of diseases has become possible [24]. The Cancer Genome Atlas (TCGA) is a landmark project that provides data on 32 human cancers gathered using genome sequencing, which has facilitated significant advancement in our understanding of the molecular basis of cancer. In this study, we conducted a large-scale bioinformatic investigation to elucidate the functional role of the SNAP25 protein in PCa.

To this end, we obtained gene expression data from the TCGA and validated the survival value of gene expression in different PCa cohorts. Due to our finding that the effect of SNAP25 protein on survival prognosis became more pronounced as the pathological grading of prostate patients became more advanced, the SNAP25 protein was validated as a prognostic biomarker and selected for further analysis. We then used integrative analysis and multiple visualization methods to explore the mechanisms of SNAP25 in PCa. We investigated the expression levels of the *SNAP25* and analyzed the correlation between the SNAP25 levels and patient clinical staging prognosis. We also used gene ontology (GO) analysis, gene set enrichment analysis (GSEA), and several methods to explore the potential functions of the *SNAP25* in tumor development and the immune microenvironment. These findings suggested a potential mechanism for SNAP25 action, and provided new insights into the important role of SNAP25 in PCa.

## Methods

### Data source and processing

Clinical and molecular data (including mRNA expression and mutations) for prostate adenocarcinoma comprising 498 tumors and 52 normal tissues in close proximity, as well as patient information, as of May 2020 were downloaded from TCGA (https://portal.gdc.cancer.gov/). The database includes mRNA expression profiles for 20,530 genes. Thereafter, genes that were differentially expressed between tumor and normal tissue were evaluated using the R package, Limma (version 3.48.3). Differentially expressed genes (DEGs) with a threshold |log_2_FC|> 2 and an adjusted p-value < 0.01 were selected for further analysis.

### Functional enrichment analysis

To reveal the biological functions of significant DEGs, Gene Ontology (GO) analysis, including analysis of the molecular functions, cellular components, and biological processes, as well as the Kyoto Encyclopedia of Genes and Genomes (KEGG) enrichment analysis were conducted with adjusted p value < 0.01 and q value < 0.01, using the R package clusterProfiler [25]. The results of the functional enrichment analysis of hub genes were visualized using the R package, GOplot [26].

### Protein–protein interaction analysis

DEGs were screened using the STRING database (https://string-db.org/) in the protein–protein interaction (PPI) network. After disregarding the disconnected nodes and screening for relevant DEGs using an interaction score > 0.4, selected PPI networks were further visualized using the Cytoscape software (version 3.8.2). The DEGs with a degree ≥ 10 were identified as hub DEGs using the cytoHubba plugin.

### Heatmap analysis

Heatmaps were visualized using the heatmap package.

### Prognostic analysis

Univariate and multivariate Cox analyses were performed using hub-DEG data and data regarding the clinical factors (age, sex, stage, and grade) of patients with PCa, obtained from TCGA, to determine the prognostic value of hub DEGs, with p-values < 0.05 in both methods considered prognostically relevant. Kaplan–Meier survival curves were plotted between the survival states and the different groups based on the median or optimal cut-off values of the gene expression levels of prognostically relevant DEGs. The primary endpoint was disease-free survival (DFS). Statistical significance was set at p < 0.05.

### TIMER database analysis

The Tumor Immune Estimation Resource (TIMER; https://cistrome.shinyapps.io/timer/) is a comprehensive web server for assessing tumor-infiltrating immune cells across different cancers [27]. TIMER includes more than 10,000 samples across the multiple cancer types in TCGA. It applies a partially deconvoluted linear least-squares regression method to work out the abundance of immune infiltrates. We assessed the correlation between SNAP25 expression and immune infiltrates, including neutrophils, CD8^+^ T cells, CD4^+^ T cells, dendritic cells, B cells, and macrophages in tumors.

### Clinical significance in TCGA

To validate the clinical relevance of prognosis determination via SNAP25, expression profiles were compared between tumors and adjacent normal tissues. Additionally, the relationship between patient information, including Gleason score, TNM stage, and the expression levels of prognostic SNAP25 were analyzed. The t-test and one-way analysis of variance (ANOVA) were used to compare two groups and multiple groups, respectively. Statistical significance was set at p < 0.05.

### GSEA analysis

To investigate the potential signaling pathways between prognostic DEGs with different expression levels, we used the TCGA-based hallmark (hallmark.all.symbols.gmt) gene set collection to carry out GSEA [28]. Nominal p-values < 0.05 and false discovery rate q-values < 0.25 were considered to be statistically significant.

### Functional experiments

#### Cell line and culture

PC3 (castration-resistant PCa) and RWPE-1 (normal prostatic epithelial) cells were purchased from Biotechnology Co (iCell Bioscience Inc, Shanghai, China). Cell lines have recently been identified using the STR method. The PC3 cells were cultured in RPMI-1640 medium (Sigma, Saint Louis, MO, USA) with 10% fetal bovine serum (FBS; Bioind, Kibbutz Beit Haemek, Israel) at 37 °C in an environment with 5% CO_2_. RWPE-1 cells were grown in Keratinocyte Serum Free Medium (iCell Bioscience Inc, Shanghai, China) at 37 °C in an environment with 5% CO_2_.

#### Western blot analysis

Total protein was extracted using RIPA lysis buffer (Beyotime, Shanghai, China) according to the manufacturer’s instructions. Subsequently, 10% sodium dodecyl sulphate (SDS)-polyacrylamide gel electrophoresis (PAGE) was performed to separate the proteins, and a western blot analysis was conducted to determine the expression of the proteins of interest, based on the experimental protocols documented in a previous study. Primary antibodies against SNAP25 (1:1000, Wanleibio, Shenyang, China) and β-actin (1:1000, ZSGB-BIO, Beijing, China) were used to probe corresponding proteins. Specific protein bands were detected with an ECL western blotting substrate (Beyotime, Shanghai, China) using the Fluorescence/Chemiluminescence Imaging System (CLINX Science instruments, Shanghai, China). and analyzed using ImageJ software. Each experiment was repeated at least three times.

#### Immunofluorescence

Immunofluorescence staining was performed to detect the expression of SNAP25 in PC3 and RWPE-1 cells. Briefly, cells were fixed with 4% paraformaldehyde for 30 min, permeabilized with 0.5% Triton X-100 for 15 min, and then blocked with goat serum. Subsequently, cells were incubated with an anti-SNAP25 (1:200, Wanleibio, Shenyang, China) antibody at 4 °C overnight, followed by incubation with an FITC-conjugated secondary antibody (ZSGB-BIO, Beijing, China) in the dark for 1 h. Cells were imaged using an immunofluorescence inverted microscope (Leica Dmi8, Wetzlar, Germany). The expression of SNAP25 was quantified using the average fluorescence intensity values generated by the ImageJ software.

#### Statistical analysis

Statistical analysis was performed primarily using ImageJ software (1.38e) and the Statistical Package for the Social Sciences (SPSS 26.0). Statistical significance was set at p < 0.05. Results were visualized using GraphPad Prism 9.2.0. All experiments were repeated three times for validation.

## Results

### DEGs in PCa was highly significant relative to that in normal prostate tissue

**Fig. 1:**
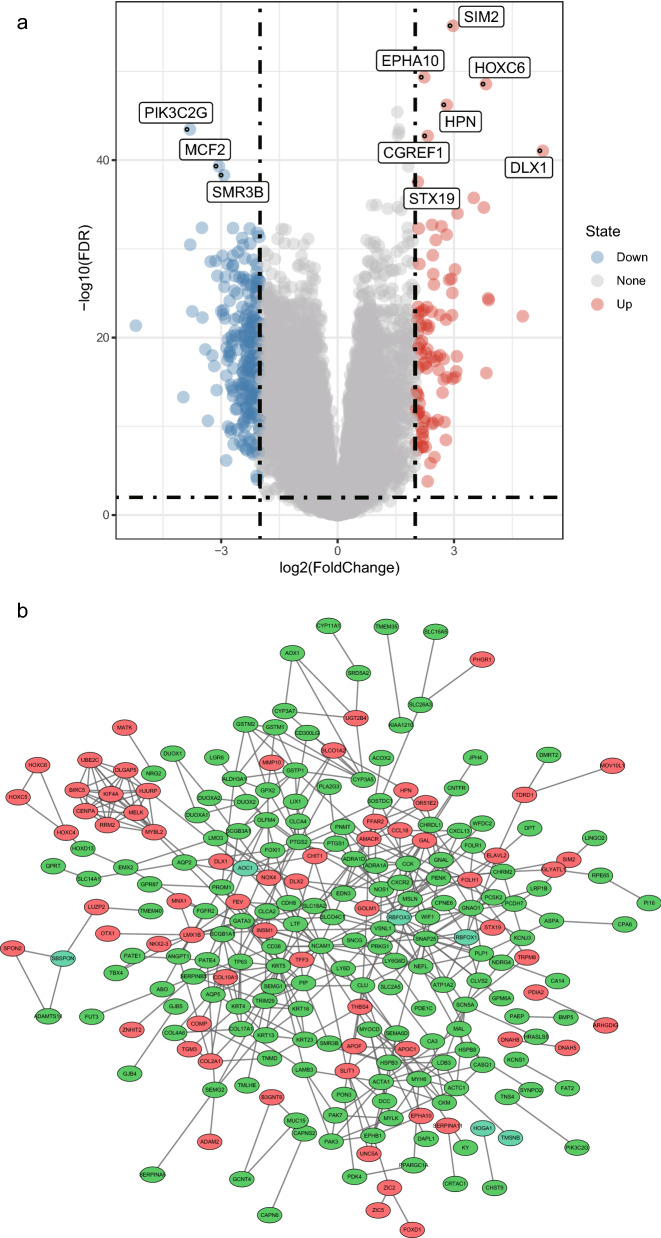
DEGs between PCa and normal prostate tissue samples. **a** Volcano plot of the DEGs between PCa and normal prostate tissue samples. **b** PPI network of DEGs was constructed using Cytoscape. Upregulated genes are marked in red; downregulated genes are marked in green

After careful screening, we selected 498 PCa samples and 52 normal prostate tissue samples, including 2394 DEGs from TCGA, for further analysis of mRNA expression profiles. Three hundred and nineteen significantly DEGs were identified using the R package, Limma, namely, 227 downregulated genes and 92 upregulated genes (|log_2_FC|> 2 and adjusted p value < 0.01). Figure 1a shows a volcano map of the 319 DEGs and Fig. 1b shows a PPI network analysis of the relationships between the DEGs, obtained using the STRING tool and Cytoscape software, including 304 nodes and 457 interactions.

### Biological function of the screened DEGs is closely related to the development of cancer

**Fig. 2:**
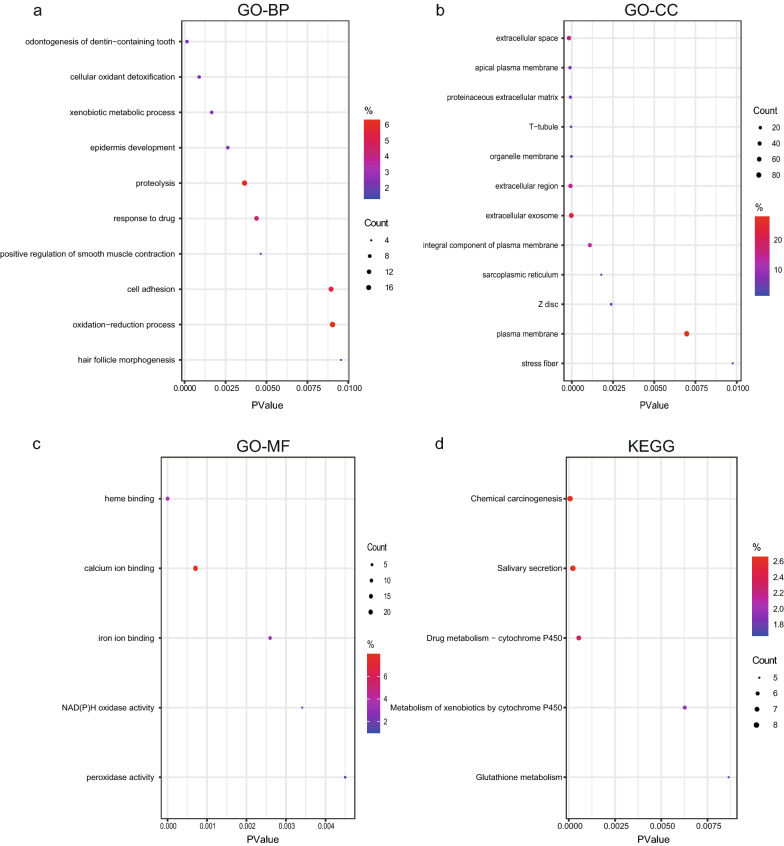
Functional enrichment analysis of differentially expressed genes. **a**–**c** GO analysis of representative DEGs, including biological process (BP), cellular component (CC), and molecular function (MF). **d** KEGG pathway terms in DEGs. The y-axis shows enriched GO and KEGG terms

To explore the biological role of differential genes, we performed GO and KEGG enrichment analyses, with the results shown in Fig. 2a, b. The main biological processes in which the DEGs were involved include cancer-related foreign body metabolism, drug response, oxidation–reduction, and cell adhesion (Fig. 2a). The cellular components closely associated with the DEGs were organelle membranes, extracellular matrix, and plasma membranes (Fig. 2b). In addition, the DEGs were significantly enriched in molecular functions related to most ion binding and enzyme activities (Fig. 2c). Analysis of the KEGG pathway demonstrated that the DEGs were mainly involved in chemotoxicity, drug metabolism, and xenobiotic activities (Fig. 2d).

### Screened hub genes are strongly associated with age and clinical stage of patients with PCa

**Fig. 3:**
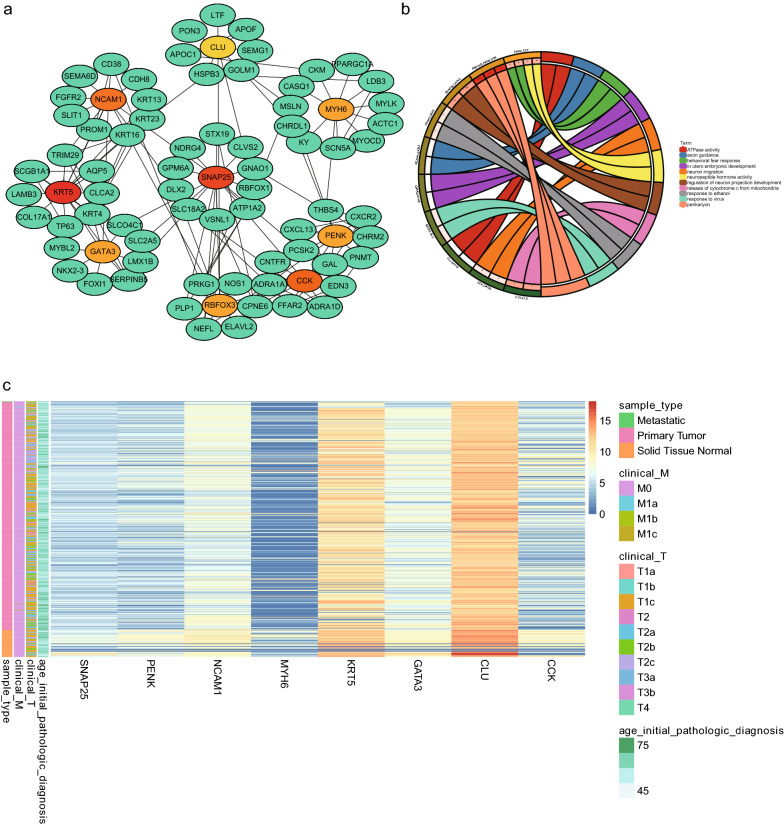
Interaction network and biological process analysis of hub DEGs. **a** Gene modules of hub DEGs. The color layout of the nine hub DEGs changed from yellow to red according to the cytoHubba plugin, indicating their increased importance. **b** GO enrichment analysis of hub DEGs. Enrichment analysis of hub genes was constructed. The number and names of hub genes involved in the gene ontology. p < 0.05 was considered statistically significant. **c** Heat map of hub DEGs. The color layout therein from blue to red indicates the expression levels of hub DEGs from down to up

To explore DEGs that play an important role in PCa pathogenesis, we performed a detailed analysis of them using the STRING database and visualized them with Cytoscape. Figure 1b represents the PPI network, including 304 nodes and 457 interactions. We then ranked hub DEGs by degree using the Cytoscape plugin cytoHubba. A gene module centered on nine hub DEGs (MYH6, SNAP25, CLU, CCK, GATA3, KRT5, NCAM1, PENK, and RBFOX3) was identified (Fig. 3a). As shown in Fig. 3b, we performed a functional analysis of the hub DEGs involved in this module using DAVID (https://david.ncifcrf.gov/), and visualized the enrichment analysis using the R package GOplot to reveal the biological roles of the hub DEGs. Moreover, the heat map showed the expression levels of pivotal DEGs (Fig. 3c). It revealed that the pivotal DEGs were different between the PCa samples and non-cancerous samples, and correlated with patient age and the clinical stage of the cancer.

### SNAP25 expression significantly correlates with prognosis in patients with pathologically graded PCa

**Fig. 4:**
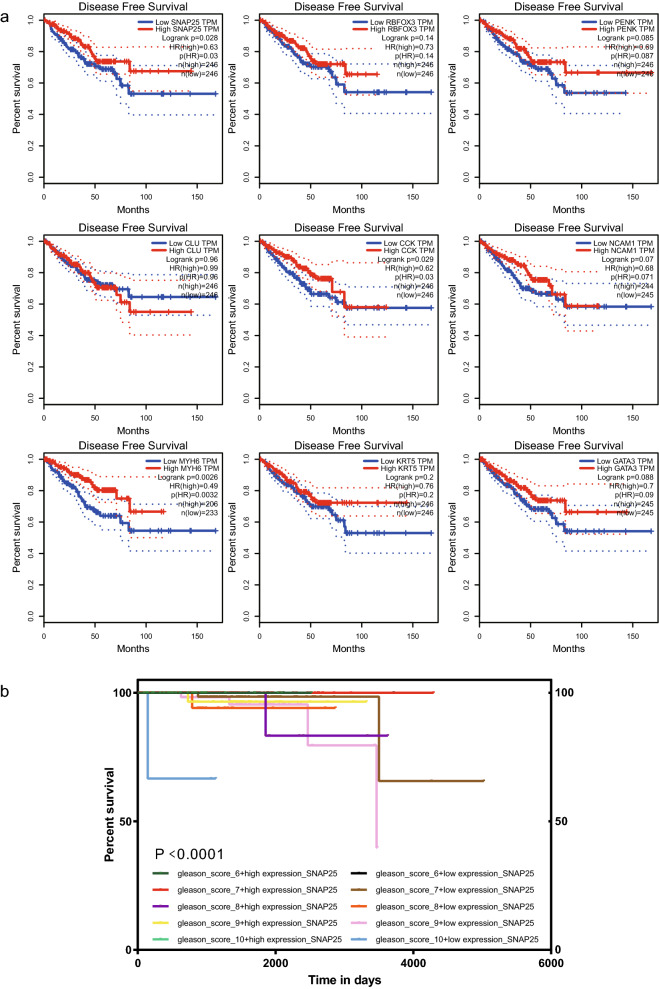
Survival curves of hub prognosis of DEGs. **a** Prognostic analysis of the training and validation datasets. relationship between hub DEGs expression in TCGA and DFS. **b** Relationship between SNAP25 expression in TCGA and OS in Gleason pathology-graded patients with PCa

To assess the independent prognostic value of hub DEGs, we extracted clinical factors (age, sex, stage, and grade) of patients with PCa in the TCGA cohort for univariate and multivariate Cox analyses. SNAP25, MYH6, and CCK were identified as significant factors affecting DFS in the model. Survival curves highlighted that low expression of SNAP25, MYH6, and CCK was significantly associated with poor DFS (p < 0.05, Fig. 4a). Furthermore, the expression status of SNAP25 was significantly associated with prognosis in patients with pathologically graded PCa (p < 0.0001, Fig. 4b). This suggests that SNAP25 can be considered a prospective biomarker for PCa and, to our knowledge, has not been previously reported as such. Thus, SNAP25 was selected for further analysis.

### Low SNAP25 expression is associated with aggravated clinical presentation in PCa

**Fig. 5:**
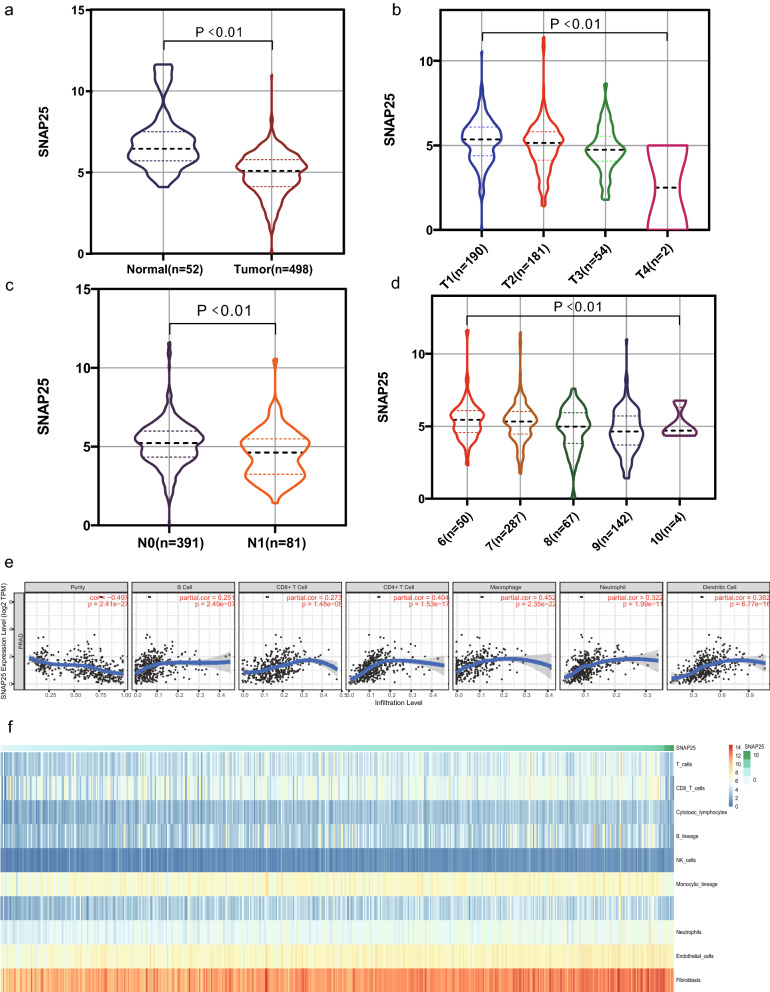
Correlation between SNAP25 expression and clinical manifestations, and tumor-infiltrating immune cells of PCa. **a** Expression of SNAP25 between cancer and normal samples. The relationship between SNAP25 expression and T-stage (**b**), N-stage (**c**) and pathological Gleason score (**d**). **e** SNAP25 expression was significantly negatively correlated with tumor purity and positively correlated with the abundance of several immune cell types in PCa. This included CD8^+^ T cells, CD4^+^ T cells, macrophages, B cells, neutrophils, and dendritic cells. **f** A heatmap of the correlation between SNAP25 and T cells, B lineage, monocytic lineage, myeloid dendritic cells, cytotoxic lymphocytes, and natural killer cells. Higher SNAP25 expression was associated with more immune infiltration in PCa

To gain more insight into the relationship between SNAP25 and the clinical presentation of PCa, we first analyzed its mRNA expression levels in tumor and normal prostate tissue samples. SNAP25 mRNA was expressed at significantly low levels in PCa cells (p < 0.01; Fig. 5a). We next explored the relationship between clinical TNM staging of PCa and SNAP25 expression. As shown in Fig. 5b, SNAP25 expression decreased as patients progressed through T stage, with low expression of SNAP25 corresponding to the late T stage (p < 0.01). Samples with stage N1 had lower SNAP25 expression than those with stage N0 (p < 0.01; Fig. 5c). As the examination of M-stage distant metastases in PCa are rarely performed clinically, too few M1-stage samples are available to rigorously assess a correlation with SNAP25. In addition, SNAP25 expression was negatively correlated with the clinicopathological Gleason score (p < 0.01; Fig. 5d), decreasing as the Gleason score increased from 6 to 10. Based on these results, SNAP25 mRNA expression was found to be clinically significant for PCa.

### SNAP25 affects the expression of immune cell populations in PCa

Based on the TIMER database, SNAP25 was closely associated with tumor-infiltrating immune cells in a variety of cancer types, including colon cancer, hepatocellular liver cancer, lung squamous cell carcinoma, testicular cancer, and PCa (Additional file 1: Figure S1). Of these, we found that SNAP25 had the most important influence on tumor-infiltrating immune cells in PCa (Fig. 5e). SNAP25 expression was negatively correlated with the purity of PCa (r = − 0.497, p < 0.01) and positively correlated with the abundance of several immune cell types, including B cells (r = 0.251, p < 0.01), CD4^+^ T cells (r = 0.404, p < 0.01), CD8^+^ T cells (r = 0.273, p < 0.01), neutrophils (r = 0.322, p < 0.01), macrophages (r = 0.452, p < 0.01), and dendritic cells (r = 0.382, p < 0.01). To verify these findings, we evaluated the relationship between SNAP25 expression and tumor-infiltrating immune cells derived from gene expression data using the MCPcounter R package to quantify the abundance of immune cells. SNAP25 expression showed a strong positive relationship with myeloid dendritic cells, T cells, monocyte lineage, B lineage, cytotoxic lymphocytes, and natural killer cells (Fig. 5f). Thus, SNAP25 may play a key role in the immune regulation of prostate tumors.

### SNAP25 promotes the migration of PCa immune cells through a linear correlation with chemokines and chemokine receptors

**Fig. 6:**
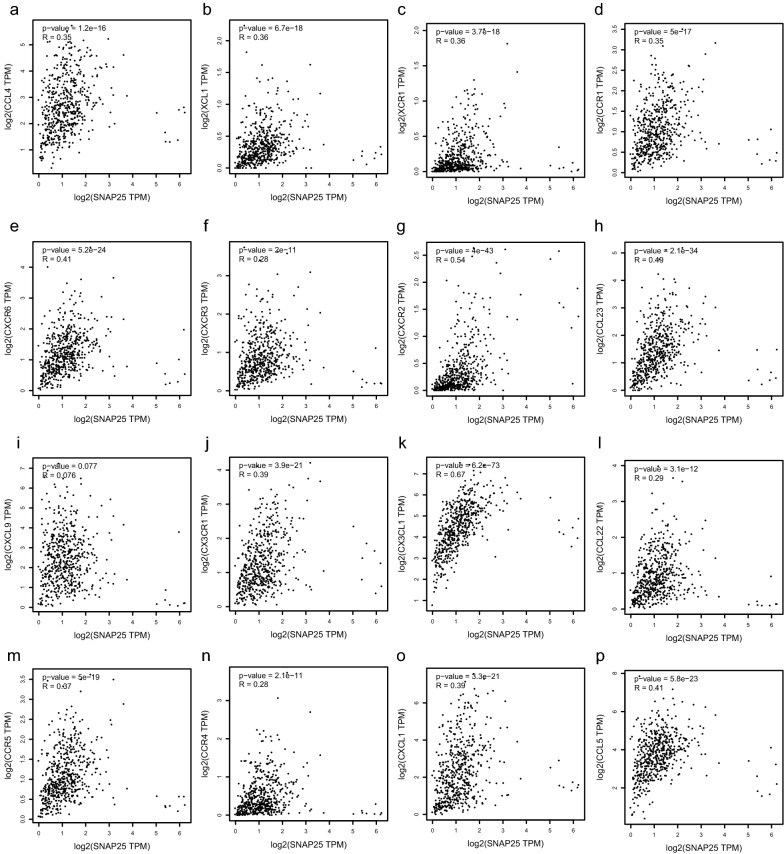
Scatter plots of SNAP25, chemokines and chemokine receptors. **a**–**p** SNAP25 was positively correlated with CCL4, CCL5, CCL22, CX3CL1, CCR1, CCR5, CX3CR1, CCL23, CXCL1, CXCR3, CXCL9, CXCR6, XCR1, CCR4, XCL1 and CXCR2

To further explore the relationship between SNAP25 and immune cell migration in PCa, we incorporated chemokines and chemokine receptors into the analysis [29], as shown in Fig. 6a–p. We discovered that SNAP25 expression was closely correlated with that of chemokines and chemokine receptors (p < 0.01), including CX3CL1, CX3CR1, CCL4, CCR5, CCL22, CCR4, CCL23, XCL1, XCR1, CXCL9, CXCR3, CXCL1, CXCR2, CXCR6, CCL5, and CCR1. The correlation images of the two different y-axes were obtained using GEPIA (http://gepia.cancer-pku.cn/). These chemokines and chemokine receptors appeared to be upregulated with increased SNAP25 expression levels. This implies that high expression of SNAP25 may facilitate the migration of immune cells to prostate tumor tissue.

**Fig. 7:**
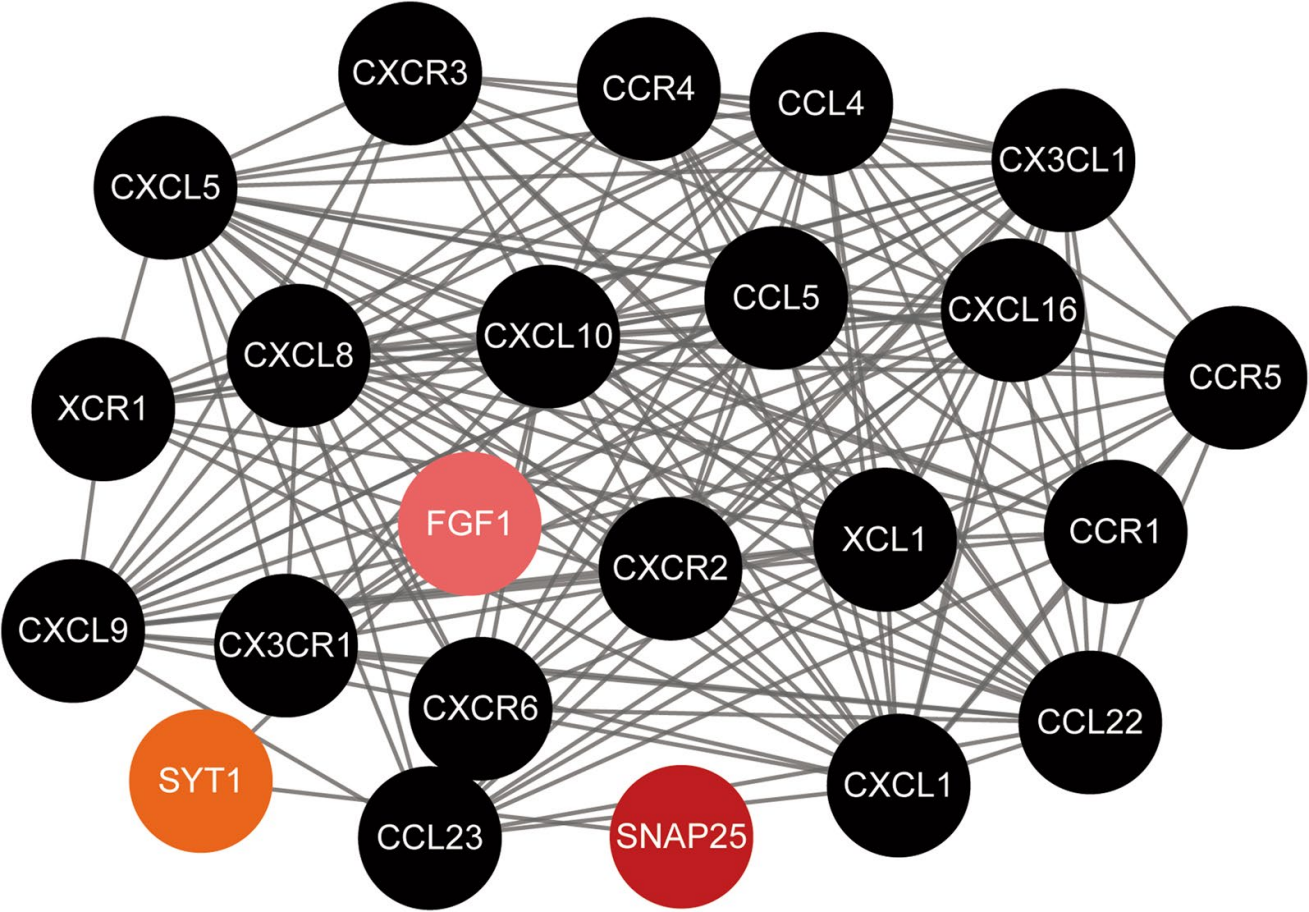
Protein–protein interaction (PPI) network based on SNAP25 and chemokine/chemokine receptor with a minimum required interaction score > 0.4. PPI network between SNAP25 and 12 chemokines and 8 chemokine receptors

To better understand the interactions between SNAP25 and chemokines or chemokine receptors, we used the STRING database for PPI network analysis and visualized them using Cytoscape. The PPI network shown in Fig. 7 indicated that SNAP25 has known or predicted interactions with the chemokines mentioned above, as well as chemokine receptors through the linkage of the FGF1 and SYT1 proteins. Chemokine, chemokine receptor, and SNAP25 interactions were extracted from the STRING database and then collated for visualization using the Cytoscape tool. These results indicate that SNAP25 may be involved in the migration of immune cells in prostate tumor tissues by regulating the expression of the FGF1 and SYT1 proteins.

### SNAP25 expression is significantly reduced at the cellular level in PCa

**Fig. 8:**
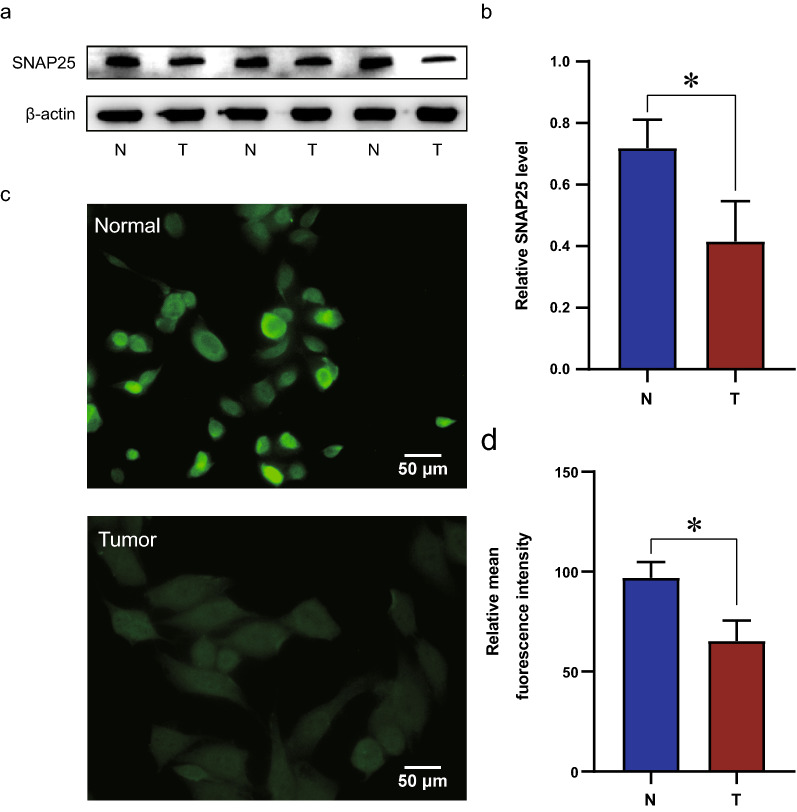
External validation of SNAP25 in molecular experiments. **a** Western blot analysis of SNAP25 between PCa and normal clinical samples. β-actin was used as an internal control. **b** Quantification of SNAP25 protein levels. (**c**) Immunofluorescence analysis of SNAP25 (green) in normal prostate cells and PCa cells. Scale bar, 50 μm. **d** The relative mean fluorescence in each group was calculated. Data were presented as the means ± SD, n = 3. *p < 0.05, *p < 0.01, and ns, p > 0.05, no significant difference

Except for validating the role of SNAP25 in TCGA datasets, the expression level of SNAP25 in PCa cells and normal prostate cells was examined by western blotting. Consistent with results using TCGA data, SNAP25 expression at the protein level was significantly lower in the tumor group than in the normal group (Fig. 8a, b). Next, we performed immunofluorescence experiments and observed a significant decrease in the expression level of SNAP25 in cancer cells (Fig. 8c, d). These results showed that decreased expression of SNAP25 predicted a poor prognosis for PCa.

### SNAP25 is associated with several tumor and immune-related pathways in PCa

**Fig. 9:**
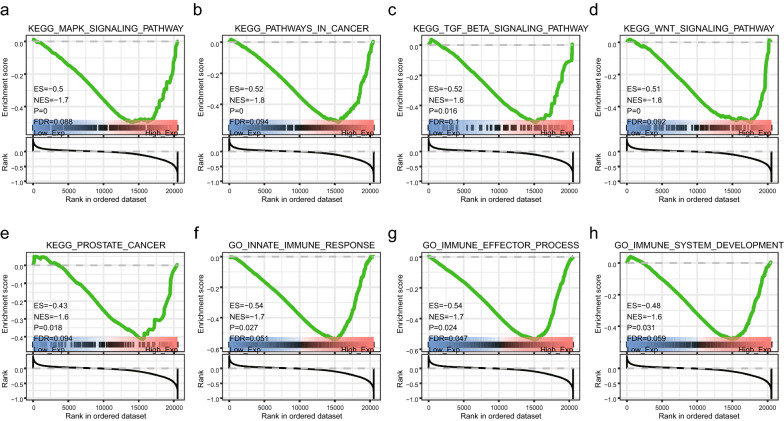
Analysis of important signaling pathways associated with SNAP25 by GSEA. **a**–**e** The most important KEGG signaling pathways associated with down-regulated SNAP25. **f**–**h** The most important GO biological processes associated with down-regulated SNAP25

GSEA was performed using samples with high and low SNAP25 expression levels to examine the pathways of enrichment. In the marker genome, downregulation of SNAP25 was clearly involved in cancer pathways, prostate cancer, and the WNT, TGF-β, and MAPK signaling pathways (Fig. 9a–e). Downregulation of SNAP25 was also involved in immune effector processes, immune system development, and the innate immune response (Fig. 9f–h). Thus, these enriched pathways are not only closely associated with prostate carcinogenesis, but also have a very important regulatory role in tumor immunity.

## Discussion

PCa is one of the most lethal genitourinary tumors in male individuals [30]. In this study, bioinformatic analysis of TCGA data was conducted to screen for the distinct genetic characteristics between PCa and normal tissue samples, resulting in the identification of the SNAP25 protein as prognostically significant. SNAP25 was expressed at low levels in PCa and positively correlated with the Gleason score and TNM stage. SNAP25 may also be involved in the biological process by which immune cells enter tumor tissue and improve the TME, thereby influencing the development of PCa and patient prognosis. In addition, we performed molecular experiments to verify the differential expression of SNAP25 in PCa and normal samples. Thus, SNAP25 was found to be a potential immune-related biomarker for PCa. Our findings provide deeper insights into the mechanisms of SNAP25 in PCa.

Using a threshold of |log_2_FC|> 2, we identified DEGs that were associated with GO terms and the KEGG pathway. Our GO pathway analysis showed that in terms of molecular function, ion binding, calcium binding, and peroxide activation are closely associated with PC. For example, multiple ion-binding channels have been reported to mediate intracellular calcium regulation associated with the hallmarks of PCa pathophysiology, including enhanced proliferation, survival, and invasion of cancer cells [31]. In terms of biological processes, differential genes are involved in cell adhesion [32], oxidation–reduction [33], and other processes that are closely related to PCa. Moreover, results of cellular component analysis mainly focused on organelles involved in biosynthesis, cellular exosomes, and extracellular compartments. For example, mitochondrial dysfunction, abnormal organelle membrane potential [34], and exosome action [35] severely affect PCa cells. In addition, KEGG pathway analysis revealed that xenobiotic metabolism, cytochrome P450 metabolism of xenobiotics, and chemical carcinogenesis were significantly associated with PCa [36]. In particular, a variety of endogenous and exogenous compounds can alter the expression levels of cytochrome P450 in humans, which in turn affects prostate cell metabolism and facilitates PCa development [37]. We further screened the dataset for important hub genes and observed that some of them showed a meaningful chance of survival based on Cox regression analysis. In addition, we found that the *SNAP25* among them significantly influenced the survival prognosis of Gleason pathology grading in prostate patients for the first time. This has not been previously reported in PCa. Therefore, we focused on SNAP25 in PCa for further studies.

In addition to its neurological aspects, SNAP25 research in oncology has received considerable attention in recent years. Recent studies have claimed that neural signaling is of great importance in regulating tumor metabolism and have validated the role of this biological function in regulating SNAP25 expression in gastric cancer metabolism [19]. Therefore, we performed a clinical relevance analysis of the *SNAP25* in patients with PCa using the TCGA database. Results showed that development of the T-stage in patients with PCa was negatively correlated with the expression of SNAP25. Patients with stage N1 had lower SNAP25 expression than those with stage N0 disease. In addition, as the Gleason score increased from 6 to 10, the expression of SNAP25 decreased.

In recent years, the tumor microenvironment and immune evasion have received significant attention in the diagnosis and treatment of a variety of malignancies. Although the immune response is heterogeneous, immunotherapy has revolutionized the field of therapeutic oncology. Studies have shown that the tumor microenvironment, particularly the immune microenvironment of tumors, is important for the prognostic outcome of patients with advanced prostate disease [38, 39]. However, few reports have described the function of SNAP25 in the TME. Using the TIMER, we found that SNAP25 affected tumor-infiltrating immune cells in PCa. SNAP25 may promote the infiltration of various immune cells in PCa, including B cells, macrophages, dendritic cells, NK cells, neutrophils, and CD8^+^ T cells. The MCPcounter method also verified that SNAP25 was positively correlated with the immune infiltration of various types of T cells, B cells, macrophages, NK cells, monocyte lines, neutrophils, fibroblasts, as well as other cells. Activation of NK cells and CD8^+^ T cells promotes anti-tumor immunity through massive secretion of various cytokines and release of perforin and granzyme [40]. Increasing infiltration of B cells contributes to tumor elimination by engaging in antigen-dependent cell-mediated cytotoxicity [41]. DCs are the most potent antigen-presenting cells, and their infiltration can help present tumor-associated antigenic peptides, or tumor-specific antigens to T cells [42]. Neutrophils can directly kill tumor cells by releasing reactive oxygen species, promote T cell activation, and recruit pro-inflammatory (M1) macrophages, anti-inflammatory (M2) macrophages, and T regulatory cells [43]. Tumor-associated macrophages [44] have been reported to play a dual role in tumor development. M1 macrophages secrete pro-inflammatory cytokines and are involved in antigen presentation and immune monitoring, whereas M2 macrophages express inhibitory cytokines. Furthermore, activated fibroblasts are important in recruiting immune cells and regulating tumor immunity [45]. Thus, SNAP25 may play a key role in the regulation of TME in PCa by participating in cellular and humoral immunity and stimulating anti-tumor function.

It is well known that chemokines control the localization and migration of immune cells. Chemokines are critical for the movement and homeostasis of immune cells [46]. We have analyzed the association between SNAP25 and chemokines/chemokine receptors to explore the potential mechanisms associated with SNAP25 in immune cell migration of PCa. The CX3CL1/CX3CR1 interactions is involved in the activation of cytotoxic T lymphocytes and NK cells and is associated with the recruitment of T cells, NK cells and monocytes [47]. The CCL4/CCR5 interaction promotes the recruitment of T cells, DCs, monocytes, and NK cells [48, 49], while CCL22/CCR4 and XCL1/XCR1 interactions play an important role in the recruitment of T cells and NK cells [50, 51]. CCL5/CCR1 is involved in macrophage and NK cell migration as well as T cell-DC interaction [52]. The CCL23/CCR1 interaction affects the migration of monocytes, neutrophils, and T cells, while CXCL9/CXCR3 facilitates recruitment of effector T cells [53]. Finally, CXCL1/CXCR2 and CXCL16/CXCR6 are related to neutrophil recruitment and natural killer T cell migration, respectively [54, 55]. In the present study, SNAP25 was found to positively correlate with the above listed chemokine/chemokine receptors, and it also has known or predicted interactions with them through the regulation of SYT1 and FGF1, according to the PPI network. This suggests that SNAP25 may increase immune infiltration in PCa by regulating the migration of immune cells in the tumor microenvironment.

Although the clinical significance and function of SNAP25 in PCa have not been reported previously, the present study demonstrates its potential as a prognostic biomarker. Interestingly, results of GSEA showed that SNAP25 was involved in many biological processes associated with PCa, including the WNT, MAPK, and TGF-β signaling pathways. In addition, SNAP25 was also involved in immune and metabolic processes, such as immune effector processes, immune system development, and innate immune responses. These results suggest that SNAP25 improves the immune response in PCa through multiple pathways.

This study includes several inherent limitations. First, although the study utilized a comprehensive bioinformatic analysis to elucidate potential functions of SNAP25, it did not explore specific signaling pathways associated with SNAP25 in PCa. In addition, in-depth molecular experiments are needed to validate the more profound mechanisms of SNAP25 and its impact on clinical outcomes in PCa. Furthermore, a tight integration and elaboration of the link between SNAP25 and chemokines/chemokine receptors could facilitate a better understanding of the important relationship between SNAP25 and the immune microenvironment in tumors.

## Conclusion

*SNAP25* is a microenvironment-related and immune-related gene that predicts a poor prognosis in PCa. Bioinformatic analysis showed that SNAP25 was not only closely associated with the clinical manifestations of PCa, but was also involved in cancer-related signaling pathways as well as immune and metabolic processes, which might provide new targets for studying the underlying mechanisms of PCa.

## Supplementary Information

**Additional file 1**: **Figure S1.** SNAP25 expression was associated with tumor purity as well as several immune cell types within different tumors. These included CD8^+^ T cells, CD4^+^ T cells, macrophages, B cells, neutrophils, and dendritic cells.

## Data Availability

The data that support the findings of this study are available in TCGA at https://portal.gdc.cancer.gov, reference number TCGA-PRAD.

## Abbreviations

SNAP25: Synaptosome associated protein 25
TCGA: The cancer genome atlas dataset
PCa: Prostate cancer
TME: The tumor microenvironment
GO: Gene ontology
KEGG: Kyoto encyclopedia of genes and genomes
GSEA: Gene set enrichment analysis
DEGs: Differently expressed genes
TIMER: The tumor immune estimation resource
FBS: Fetal bovine serum
PPI: Protein–protein interaction
DFS: Disease-free survival
OS: Overall survival
