# Supplementary figures and images for: SNAP25 is a potential prognostic biomarker for prostate cancer
Source: Cancer Cell Int. 2022 Apr 7;22:144. doi: 10.1186/s12935-022-02558-2 (PMC8991690; doi:10.1186/s12935-022-02558-2)

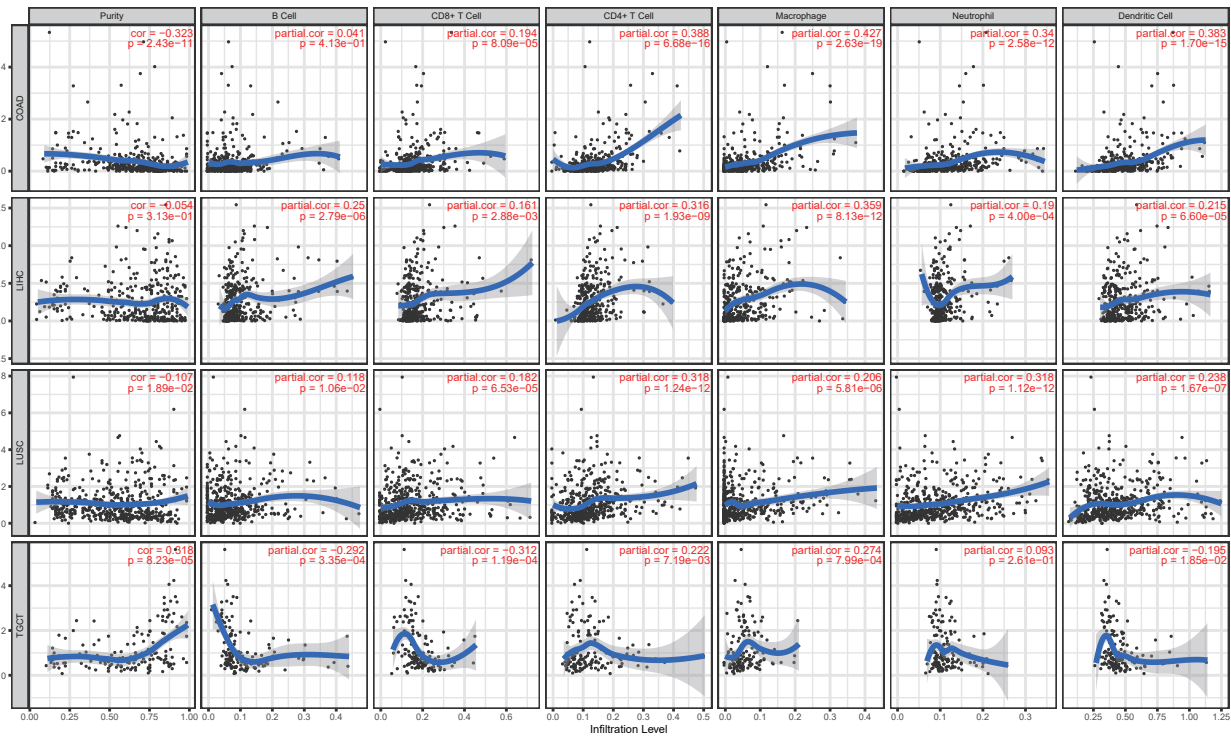

Supplement: Supplementary file 1 — Additional file 1: Figure S1. SNAP25 expression was associated with tumor purity as well as several immune cell types within different tumors. These included CD8+ T cells, CD4+ T cells, macrophages, B cells, neutrophils, and dendritic cells. [file 12935_2022_2558_MOESM1_ESM.pdf]
